# Iron-Dependent Trafficking of 5-Lipoxygenase and Impact on Human Macrophage Activation

**DOI:** 10.3389/fimmu.2019.01347

**Published:** 2019-06-28

**Authors:** Beatrice Dufrusine, Andrea Di Francesco, Sergio Oddi, Lucia Scipioni, Clotilde Beatrice Angelucci, Claudio D'Addario, Mauro Serafini, Ann-Kathrin Häfner, Dieter Steinhilber, Mauro Maccarrone, Enrico Dainese

**Affiliations:** ^1^Faculty of Bioscience and Technology for Food Agriculture and Environment, University of Teramo, Teramo, Italy; ^2^Faculty of Veterinary Medicine, University of Teramo, Teramo, Italy; ^3^European Center for Brain Research (CERC)/IRCCS Santa Lucia Foundation, Rome, Italy; ^4^Institute of Pharmaceutical Chemistry, Goethe-University Frankfurt, Frankfurt, Germany; ^5^Department of Medicine, Campus Bio-Medico University of Rome, Rome, Italy

**Keywords:** 5-lipoxygenase, macrophages, iron, enzyme activation, nuclear translocation, macrophage activation

## Abstract

5-lipoxygenase (5-LOX) is a non-heme iron-containing dioxygenase expressed in immune cells that catalyzes the two initial steps in the biosynthesis of leukotrienes. It is well known that 5-LOX activation in innate immunity cells is related to different iron-associated pro-inflammatory disorders, including cancer, neurodegenerative diseases, and atherosclerosis. However, the molecular and cellular mechanism(s) underlying the interplay between iron and 5-LOX activation are largely unexplored. In this study, we investigated whether iron (in the form of Fe^3+^ and hemin) might modulate 5-LOX influencing its membrane binding, subcellular distribution, and functional activity. We proved by fluorescence resonance energy transfer approach that metal removal from the recombinant human 5-LOX, not only altered the catalytic activity of the enzyme, but also impaired its membrane-binding. To ascertain whether iron can modulate the subcellular distribution of 5-LOX in immune cells, we exposed THP-1 macrophages and human primary macrophages to exogenous iron. Cells exposed to increasing amounts of Fe^3+^ showed a redistribution (ranging from ~45 to 75%) of the cytosolic 5-LOX to the nuclear fraction. Accordingly, confocal microscopy revealed that acute exposure to extracellular Fe^3+^, as well as hemin, caused an overt increase in the nuclear fluorescence of 5-LOX, accompanied by a co-localization with the 5-LOX activating protein (FLAP) both in THP-1 macrophages and human macrophages. The functional relevance of iron overloading was demonstrated by a marked induction of the expression of interleukin-6 in iron-treated macrophages. Importantly, pre-treatment of cells with the iron-chelating agent deferoxamine completely abolished the hemin-dependent translocation of 5-LOX to the nuclear fraction, and significantly reverted its effect on interleukin-6 overexpression. These results suggest that exogenous iron modulates the biological activity of 5-LOX in macrophages by increasing its ability to bind to nuclear membranes, further supporting a role for iron in inflammation-based diseases where its homeostasis is altered and suggesting further evidence of risks related to iron overload.

## Introduction

Lipoxygenases (LOXs) catalyze the regio- and stereo-specific insertion of molecular oxygen into polyunsaturated fatty acids (1). In humans, 5-LOX, 12-LOX, and 15-LOX1 constitute the most studied enzymes with specific distribution in hematopoietic cells (2). Among them, 5-LOX is of major patho-physiological relevance, since it has been implicated in the biosynthesis of important inflammatory bioactive lipid mediators (3). In particular, arachidonic acid (AA) released upon hydrolysis catalyzed by phospholipase A_2_ (cPLA_2_), serves as the 5-LOX substrate for leukotrienes (LTs) and lipoxins biosynthesis. These bioactive lipids act as hormone-like factors in biological processes and display diverse functions in the immune system serving as important molecules for effective regulatory functions in macrophages, acting in both innate immunity and adaptive response (4).

The subcellular localization of 5-LOX in unstimulated cells differs within cell types (5, 6). In peripheral blood neutrophils (7), differentiated HL-60 cells (8), and peritoneal macrophages (9), 5-LOX is mainly localized in the cytosol, whereas in resting alveolar macrophages (10), rat basophilic leukemia cells (11), bone marrow–derived mast cells (12), and Langerhans cells of human skin (13), the same enzyme is either partly or predominantly present in the soluble compartment of the nucleus. Upon stimulation, both cytosolic and nuclear 5-LOXs translocate to the nuclear envelope, leading to interaction with the 5-LOX activating protein (FLAP), a small protein localized in internal cell membranes that is essential in the functional processing of endogenous AA (3, 14). In particular, the translocation from the cytosol to the nuclear membrane of 5-LOX and its co-localization with FLAP is clearly emerging as an early and rate-limiting mechanism of activation that triggers different signaling pathways leading to the synthesis of different classes of pro-inflammatory LTs (LTA_4_ and LTC_4_) (14, 15).

In this context, the available crystal structures of LOXs indicate single polypeptide chain proteins adopting a two-domain folding: the N-terminal “C2-like” domain (~120 amino acids), which confers Ca^2+^-dependent membrane binding ability to 5-LOX, and is crucial for bringing the enzyme in proximity to its AA substrate within the nuclear membranes (16) and the larger catalytic C-terminal domain, that is primarily α-helical and harbors the non-heme catalytic iron (17). In a site-directed mutagenesis study aimed at investigating the intracellular distribution of 5-LOX, mutations known to abolish enzyme activity, and affecting the binding to iron in the active site, induced a graded distribution of 5-LOX in the nucleus and cytosol, depending on the iron content (12). Trypsin cleavage of soybean-LOX1 at Lys 277 yields a “mini-LOX” that roughly represents the catalytic subunit, with enhanced catalytic efficiency and higher membrane binding ability compared to the full-length native enzyme (18, 19). Extraction, reconstitution and substitution of iron revealed a non-catalytic role for it in modulating the membrane-binding ability of mini-LOX (20). In particular, it was shown that the correct coordination geometry of iron in the active site stabilizes an enzyme conformation that becomes more competent for the selective targeting and binding to the membrane surface, thus allowing more effective substrate recognition (20).

More recently, we have analyzed by molecular dynamics simulations the conformational changes induced by iron removal in 5-LOX indicating that the degree of enzyme flexibility is related to the presence of iron into the active site (21). These data provide further evidence on the functional role of iron in the activation of LOX, but little is known about 5-LOX activity and intracellular localization after iron exposure in innate immunity cells.

In this study we firstly studied by FRET the effect of iron removal in modulating the activity and membrane binding of human recombinant 5-LOX to synthetic membranes. Then we assessed *in vitro* the effects on membrane binding, nuclear translocation, and activity of 5-LOX of acute exposure of exogenous iron or hemin (ferriprotoporphyrin IX chloride) in THP-1 macrophages and human macrophages. We found that *in vitro* iron removal decreases membrane binding of 5-LOX and, that acute iron treatment of macrophages yields a substantial increase of 5-LOX activity and its association along with FLAP with the nuclear envelope.

## Materials and Methods

RPMI 1640 medium was from Gibco BRL (Life Technologies, Rockville, MD); fetal bovine serum (FBS), adenosine triphosphate (ATP), arachidonic acid (AA), ferric chloride (FeCl_3_), hemin, phorbol-12-myristate-13-acetate (PMA), protease inhibitor cocktail, and phenylmethylsulfonyl fluoride (PMSF) were purchased from Sigma (St. Louis, MO, USA). For immunological studies we used the following antibodies: anti-5-LOX (Becton Dickinson, Franklin Lakes, NJ, USA), anti-β-actin (Millipore, Billerica, MA, USA), anti-lamin (Santa Cruz Biotechnology, Santa Cruz, CA, USA), and anti-FLAP (Abcam, Cambridge, UK). Goat Alexa Fluor-conjugated secondary antibodies and Prolong Gold anti-fade kit were purchased from Molecular Probes (Eugene, OR, USA). Macrophage colony-stimulating factor (M-CSF) and human serum were purchased from Miltenyi Biotec (Bergisch Gladbach, Germany). All other chemicals were from Sigma Chemical Co. (St. Louis, MO, USA), unless otherwise indicated.

### Enzyme Preparations and Enzymatic Assay

Recombinant human 5-LOX was expressed in *E. coli* from the plasmid pT3-5-LOX and purified (purity was >95%, see Supplementary Figure 1) on ATP-agarose (Sigma A2767) followed by anion exchange chromatography, as previously reported (22). Apo-5-LOX enzyme was obtained by metal removal using the iron chelator deferoxamine (DFO). To this aim enzyme solutions were dialyzed overnight against 50 mM Tris/HCl pH 7.5 buffer using a 5-LOX:DFO stoichiometry of 1:5, followed by dialysis against the same buffer containing 2 mM EDTA for 48 h. All experiments were performed using iron-free water, dialysis bags, and plastics. 5-LOX activity was assayed spectrophotometrically at 25°C in 50 mM Tris/HCl pH 7.5 buffer by recording the formation of conjugated hydroperoxides from AA at 234 nm.

### Liposomes Preparations and FRET Studies

Large unilamellar vesicles mimicking the biophysical properties of nuclear membranes were prepared using 1-palmitoyl-2-oleoyl-sn-glycero-3-phosphocholine (POPC) as described previously (23). Fluorescence spectra were recorded at 25°C using a PerkinElmer LSB50 fluorimeter and 10 × 2 mm path length quartz fluorescence microcuvettes (Hellma, Concord, ON). The pyrene bound liposomes used in FRET studies contained 5% (w/w) Py-PE (1,2-dioleoyl-sn-glycero-3-phosphoethanolamine-N-1–pyrenesulfonyl) purchased from Molecular Probes. 5-LOX was used at a final concentration of 0.2 μM, whereas the liposome concentration varied between 10 and 600 μM in a final volume of 100 μL. The membrane binding measurements of both apo- and holo-5-LOX were carried out in Ca^2+^ free solutions after an incubation of the enzyme at different liposome concentrations for 5 min.

### THP-1 Macrophages

The human THP-1 cells were maintained in RPMI 1640 medium containing glutamine and supplemented with 10% FBS, 100 mg/mL streptomycin, 100 U/mL penicillin, 1 mM sodium pyruvate. For monocyte to macrophage differentiation, THP-1 cells were seeded at a density of 2–3 × 10^5^ cells/mL and treated with 100 ng/mL PMA for 2 days (24).

### Human Primary Macrophages

To obtain human macrophages, peripheral blood mononuclear cells, isolated after venous puncture from healthy donors, were cultured in 1640 RPMI medium supplemented with 10% FBS, 5% human serum, 100 U/mL penicillin/streptomycin, and differentiated with 25 ng/mL M-CSF for 6–7 days at 37°C in a humidified 5% CO_2_ atmosphere.

### Real-Time PCR Analysis

Messenger RNA was extracted from macrophages using Qiagen minikits (Qiagen, Mississauga, ON, Canada), as per manufacturer's instructions, and was quantitated spectrophotometrically. One μg of total mRNA was reverse transcribed to cDNA, using iScript^TM^ cDNA synthesis kit (Bio-Rad, Hercules, CA, USA). cDNA (50 ng) was taken for real-time PCR using iTaq^TM^ Fast SYBR® Green supermix with ROX (Bio-Rad, Hercules, CA, USA) on an DNA Engine Opticon 2 Continuous Fluorescence Detection System (MJ Research, Waltham, MA, USA). Intron-spanning primers to amplify ~200 bp were designed using Primer Express v.2.0 Software (Applied Biosystems, Foster City, CA, USA). Primer sequences were: 5-LOX forward 5′-TGCCAAATGCCACAAGGATT-3′ and reverse 5′-TGCATGAAGCGGTTGATGAA-3′; p12-LOX forward 5′-TGGTCATCCAGATTCAGCCTC-3′ and reverse 5′-TGGATCTCGTGCAGTTGGAA-3′; 15-LOX1 forward 5′-TGTGAAAGACGACCCAGAGCT-3′ and reverse 5′-TGACAAAGTGGCAAACCTGGT-3′; GADPH forward 5′-GTGAAGGTCGGAGTCAACGGA-3′ and reverse 5′-GAGGGATCTCGCTCCTGGAAGA-3′. Dissociation curve analysis following each amplification reaction was carried out to confirm the amplification of primer-specific products. All data were normalized to the endogenous reference gene glyceraldehyde-3-phosphate dehydrogenase (GAPDH). Differences in threshold cycle (Ct) number were used to quantify the relative amount of PCR targets contained in each tube. Relative amounts of different gene transcripts were calculated by the ΔΔCt method, and were converted to relative transcription ratio (2^−ΔΔ*Ct*^) for statistical analysis (25).

### LOX Activity in THP-1 Macrophages

The 5-LOX activity was assayed partially modifying the already described procedure (26). For assays of cells, THP-1 monocytes were seeded for 48 h in 96-well microtiter plates at 1 × 10^5^ cells/mL (100 μL/well) and differentiated into macrophages as described above. LOX inhibitors dissolved in DMSO (final concentration, 0.1%, v/v) were added at different concentrations to each well along with H_2_DCFDA (10 μM) and incubated for 30 min in the dark at 37°C in a CO_2_ incubator (5% CO_2_/95% air). Similarly, vehicle (DMSO) was added for control samples. The cell-permeant 2′,7′-dichlorodihydrofluorescein diacetate (H_2_DCFDA) was freshly prepared in ethanol for each assay. After careful removal of the loading medium, the cells were washed briefly with GIBCO Hanks' buffered salt solution (HBSS) (Invitrogen, CA, USA) before adding a reaction buffer containing 2.5 mM CaCl_2_, 2 mM ATP and AA (70 μM), the substrate for lipoxygenase in HBSS. After adding reaction buffer, the fluorescence product of H_2_DCFDA was analyzed using a microplate reader (Thermo Scientific, USA) for 30 min at 37° at excitation and emission wavelengths of 485 and 528 nm, respectively. The increase in fluorescence per well was calculated by the formula F_30_-F_0_, where F_30_ = fluorescence at time 30 min and F_0_ = fluorescence at time 0 min (taken immediately after adding substrate). This method avoids background fluorescence and the need to include blank wells in experiments. The percentage activity was calculated by considering fluorescence of control cells as 100% activity. All the experiments were performed at least in triplicates.

### Cell Treatments and Subcellular Fractionation by Detergent Lysis

THP-1 macrophages (1 × 10^7^ cells) were treated in the presence or absence of Fe^3+^ or hemin at indicated concentrations, at 37°C in a cell incubator (5% CO_2_/95% air). After a 5 min incubation period, the monolayers of cells were chilled on ice and briefly washed with HBSS before adding 1 mL of ice-cold NP-40-lysis buffer (10 mM Tris-HCl, pH 7.4, 10 mM NaCl, 3 mM MgCl_2_, 1 mM EDTA, 0.1% NP-40, 1 mM PMSF, 60 mg/mL soybean trypsin inhibitor, and 10 mg/mL leupeptin), kept on ice for 10 min, and gently scraped and centrifuged (800 *g*, 10 min, at 4°C). Supernatants (non-nuclear fractions) were transferred to a new tube, and pellets (nuclear fractions) were resuspended in 200 μL ice-cold relaxation buffer (50 mM Tris-HCl, pH 7.4, 250 mM sucrose, 25 mM KCl, 5 mM MgCl_2_, 1 mM EDTA, 1 mM PMSF, 60 mg/mL soybean trypsin inhibitor, 10 mg/mL leupeptin). Both nuclear and non-nuclear fractions were centrifuged again (800 *g*, 10 min, at 4°C) for further purification. Lysis of cells and integrity of nuclei were confirmed by light microscopy with trypan blue exclusion. Nuclei in relaxation buffer were disrupted by sonication (3 × 5 s). Aliquots of nuclear and non-nuclear fractions were immediately mixed with the same volume of Laemli sample buffer, heated for 5 min at 95°C, and analyzed for 5-LOX protein content by sodium dodecyl sulfate–polyacrylamide gel electrophoresis (SDS-PAGE) followed by immunoblotting. Lamin, a ubiquitous protein exclusively present in the nuclear membrane, was used as a marker of the nuclear fraction.

### Immunoblot Analysis of Subcellular Fractions

Aliquots (25 μL) of pair-wise subcellular fractions (cytosol and nucleus), corresponding to equal amounts of cells, were mixed with 4 mL glycerol/ 0.1% bromphenol blue (1:1, V/V) and analyzed by SDS-PAGE using a Mini Protean III system (Bio-Rad, Hercules, CA, USA) on a 4 to 15% linear gradient gel. After electroblotting to PVDF membrane (GE-Healthcare, Pollards Woods, UK), proteins were blocked with 5% non-fat dry milk in Tris-buffered saline in the presence of 0.1% Tween (TBS-T) for 1 h at room temperature. Membranes were washed and incubated with primary antibodies overnight at 4°C. Then, membranes were washed with TBS-T and incubated with 1:1,000 dilution of HRP–conjugated secondary antibodies (Sigma, St. Louis, MO, USA) for 1 h at room temperature. After washing with TBS-T, 5-LOX protein was visualized using the HRP substrate ECL Prime (GE-Healthcare, Pollards Woods, UK). Densitometry was performed with a Gel Doc 1000 instrument and the Molecular Analyst software (Bio-Rad, Hercules, CA, USA).

### Confocal Analysis

For assess the subcellular distribution of FLAP and 5-LOX and their co-localization, THP-1 macrophages, and human primary macrophages were plated on glass coverslips in 12-well plates. Cells were left untreated (Ctrl) or treated with 10 μM hemin or 10 μM FeCl_3_ for 5 min, fixed with ice-cold acetone for 5 min and then double stained with rabbit anti-FLAP (1:100; Abcam, Cambridge, UK) and mouse anti-5-LOX primary antibodies (1:100; Becton Dickinson). As positive control, cells were treated for 5 min with 5 μM A23187, a Ca^2+^ ionophore that is successfully used to stimulate the translocation of 5-LOX to the nuclear envelop. After incubation with the cocktail of primary antibodies for 24 h, samples were incubated for 1 h at room temperature in a mixture of secondary antibodies including Alexa Fluor 488-conjugated goat anti-rabbit IgG (1:200; Molecular Probes) and Alexa Fluor 568-conjugated goat anti-mouse IgG (1:200; Molecular Probes). Cells were then DAPI (Sigma) counterstained, air-dried and coverslipped with Prolong Gold anti-fade. Images were acquired with an Ultraview Vox Spinning Disk (PerkinElmer, Milan, Italy) equipped with a 63 × 1.4-NA Plan-Apochromat oil immersion objective and an EMCCD C9100-50 camera. FLAP and 5-LOX fluorescence intensities were calculated through the NIH ImageJ software on 20 cells in different fields of two independent experiments. Overlap coefficient was measured by using JACOP plugin of ImageJ software. Apparent co-localization due to random staining, or very high intensity, in one window will have values of overlap coefficient near to zero, while if the two signal intensities are interdependent (co-localized) these values will be positive with a maximum of 1. We restricted the co-localization analysis to strip-like (curvilinear) regions-of-interest, which were 1.7 μm in width and had contour lengths from 10 to 70 μm, circumscribing part or all of the nuclear envelope. Thresholds were not set by the operator, but automatically calculated by the software to avoid biased data. All microscope quantifications shown in the article were performed by a blind approach. For presentation purposes, pictures were exported in TIFF format and processed with Adobe Photoshop CS5 (Adobe), for adjustments of brightness and contrast.

### Functional Analysis of THP-1 Macrophages by ELISA Assay

THP-1-derived macrophages were treated in the presence or absence of 10 μM Fe^3+^ for 5 min, 10 μM hemin for 5 min, and 20 μM DFO for 2 h, before 10 μM hemin exposure. After treatments the medium were replaced, and the supernatants collected after 24 h. The concentration of interleukin-6 (IL-6) in the cellular supernatants were determined using the Human IL-6 Uncoated Invitrogen ELISA Kit assay (ThermoFisher, San Diego, USA) applying the manufacturer's directions. The plates were read at 450 nm and the sensitivity of the used ELISA assay was in the range 2–200 pg/mL.

### Statistical Analysis

Data reported in this paper are the mean ± SE of at least three independent experiments, each performed in triplicate. For each experimental setting, data are expressed as percentage of the control value of that specific experiment. A treatment was significant when *p* was <0.05 by analysis of variance, and subsequently by Student's unpaired two-tailed *t*-test in the Prism 5 program (GraphPAD Software for Science, San Diego, CA, USA).

## Results

### Membrane Binding Properties of Holo- and Apo-5-LOX

**Figure 1:**
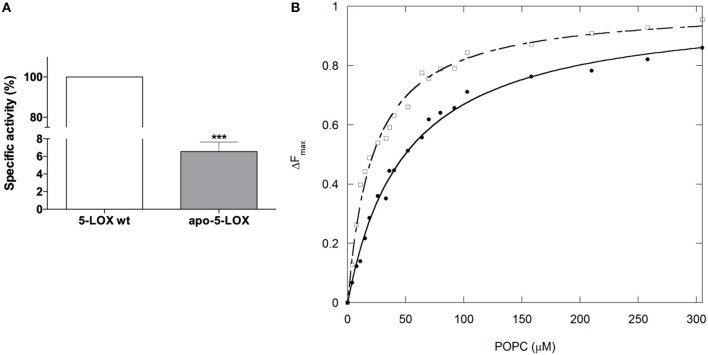
Iron removal impairs activity and reduces membrane binding of 5-LOX. **(A)** Generation of apo-5-LOX by iron chelation leads to a significant reduction (~90%) of its enzymatic activity (100% of holo-5-LOX form specific activity corresponding to 225.15 ± 6.18 μmol/min per mg of protein). **(B)** The binding isotherm analyzed as tryptophan FRET quenching at different concentrations of POPC liposomes demonstrated a significant decrease of the membrane affinity of apo- (filled circles) with respect to holo-5-LOX (empty squares). (^***^p < 0.001 vs. 5-LOX wt).

We recently reported an unprecedented role for iron in modulating catalytic activity, structural stability, and membrane binding properties of soybean LOX-1 (20), supporting the notion that iron is not only essential for maintaining a proper structural integrity of the enzyme activity but also for its membrane association. To assess whether these effects can be also extended to the human LOXs, and to gain insight into the mechanism(s) by which iron may modulate 5-LOX membrane binding, we investigated by FRET the membrane binding properties of apo-5-LOX and holo-5-LOX. As expected, the apo-5-LOX obtained by iron removal led to an almost complete loss of its enzymatic activity (~95% decrease) (Figure 1A). More interestingly, we found that iron removal induced a significant decrease (~2-fold over the holo-5-LOX) in the affinity of the apo-form of the enzyme for POPC membranes, as indicated by an increase in [L]_1/2_ (49.4 ± 2.8 μM) of the apo-enzyme with respect to the value calculated for holo-5-LOX (Figure 1B).

### LOX Activity in THP-1 Macrophages Increases After Acute Fe^3+^ Exposure in a Concentration-Dependent Manner

**Figure 2:**
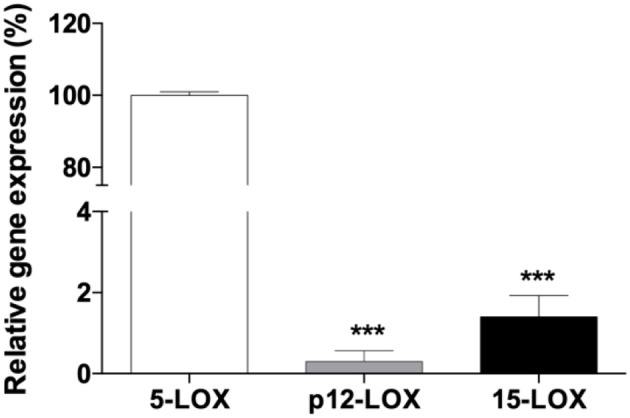
Quantitative real-time PCR analysis of 5-LOX, p12-LOX, and 15-LOX1 mRNAs in human THP-1 macrophages. The gene of 5-LOX was the most expressed in PMA-differentiated macrophages. GAPDH was used as an endogenous control, and the expression of LOX isoforms was represented using 5-LOX as calibrator. Each bar represents the mean ± SE of three independent experiments (^***^p < 0.001 vs. 5-LOX).

**Figure 3:**
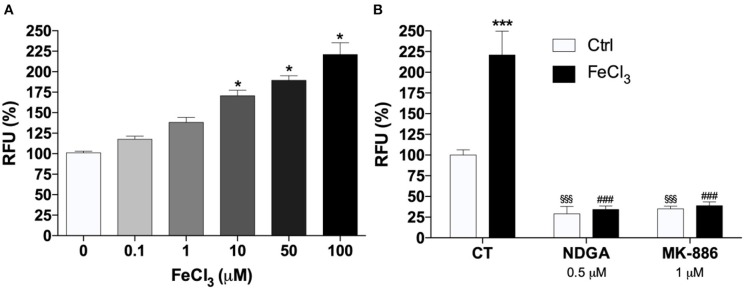
Iron increase 5-LOX activity. **(A)** Effect of exogenous iron (FeCl_3_) on 5-LOX activity tested by a cell-based fluorescence assay. To determine the effect of Fe^3+^ on 5-LOX activity different concentrations of FeCl_3_ were added to each well and incubated for 5 min in the dark at 37° in a CO_2_ incubator (5% CO_2_/95% air). Enzyme assays were started by the addition of 2.5 mM CaCl_2_, 2 mM ATP, and 70 μM AA (^*^P < 0.05 vs. control). **(B)** Pharmacological blockade of 5-LOX activation prevented induction of 5-LOX activity by FeCl_3_. THP-1 macrophages were incubated with 0.5 μM NDGA or 1μM MK-886 for 30 min and then treated with 1 mM FeCl_3_ or vehicle. The results represent the means of RFU ± SE of three independent experiments. relative to the control activity (no FeCl_3_) assumed as 100% (^***^p < 0.001 vs. control; ^§§§^*p* < 0.001 vs. control; while ^*###*^*p* < 0.001 vs. control with FeCl_3_).

Real-time PCR analysis of *5-LOX, 12-LOX*, and *15-LOX* revealed that *5-LOX* was the most expressed gene in THP-1 macrophages (Figure 2). In particular ΔCt values, normalized to GAPDH levels, were as follows: *5-LOX* 7.15 ± 0.18; *12-LOX* 19.75 ± 1.85; *15-LOX* 13.73 ± 1.66. LOX activity was assayed in THP-1 macrophages with different AA concentrations (from 25 to 100 μM). Seventy μM was found to be the optimal concentration for the cellular assay (data not shown). Cells exposed to increasing concentrations of Fe^3+^ for 5 min, and then stimulated with 70 μM AA, showed a dose-dependent increase of LOX activity. Fe^3+^ at concentrations from 10 to 100 μM led to a significant 1.5- to 2-fold increase of enzyme activity (Figure 3A). Pre-incubation with NDGA (0.5 μM) or MK-886 (1 μM), a FLAP inhibitor, completely reversed the effect of Fe^3+^ (Figure 3B).

### Acute Fe^3+^ Exposure Induces 5-LOX Nuclear Translocation in THP-1 Macrophages

**Figure 4:**
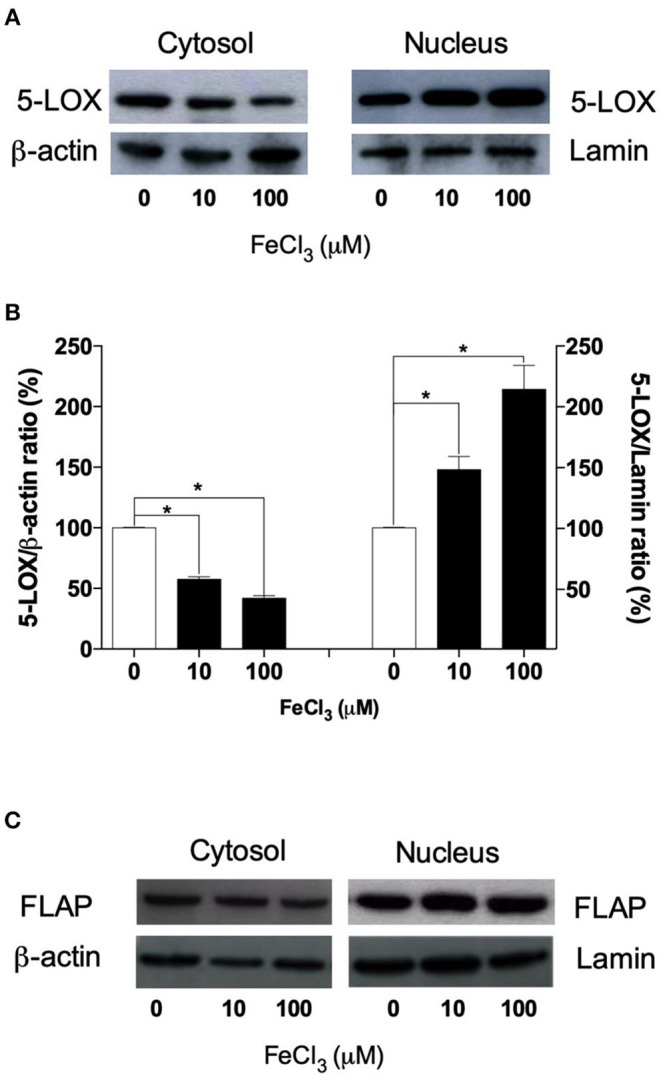
Western blot analysis of 5-LOX in subcellular fractions from THP-1 macrophages. **(A)** 5-LOX protein was found predominantly in the nuclear fraction of resting THP-1 macrophages. However, priming cells with exogenous Fe^3+^ (10–100 μM) for 5 min led to a redistribution of the cytosolic 5-LOX to the nuclear compartment. **(B)** The relative intensities of the same bands shown in **(A)** were determined by densitometry (arbitrary units, AU). Similar results were obtained in two additional experiments (not shown). The results are given as mean ± SE of three independent experiments (^*^p < 0.05 vs. control). **(C)** The expression and cellular localization of FLAP were not altered by iron treatment. β-actin and lamin were used as housekeeping proteins for data normalization.

To ascertain whether Fe^3+^ can modulate the 5-LOX translocation from cytosol to nuclear envelope, we assessed the localization of 5-LOX by means of subcellular fractionation, using a lysis buffer containing the NP-40 detergent (0.1%) and 5-LOX immunoblotting. This technique yields a nuclear fraction with intact nuclei, and a non-nuclear fraction containing cytosol, plasma membrane, endoplasmic reticulum, Golgi apparatus, and cytoskeletal proteins (11). In untreated THP-1 macrophages, 5-LOX protein was found both in the cytoplasm and nucleus fractions (Figures 4A,B). Acute exposure of cells with exogenous Fe^3+^ (10–100 μM) for 5 min led to a significant redistribution (from ~45 to 75%; *p* < 0.05) of the cytosolic 5-LOX to the nuclear envelope, as determined by densitometric analysis (Figure 4B). By contrast, the expression and cellular localization of FLAP were not altered by Fe^3+^ treatment (Figure 4C).

### Acute Hemin Exposure Recapitulates the Effects of Fe^3+^ on 5-LOX Translocation in THP-1 Macrophages

**Figure 5:**
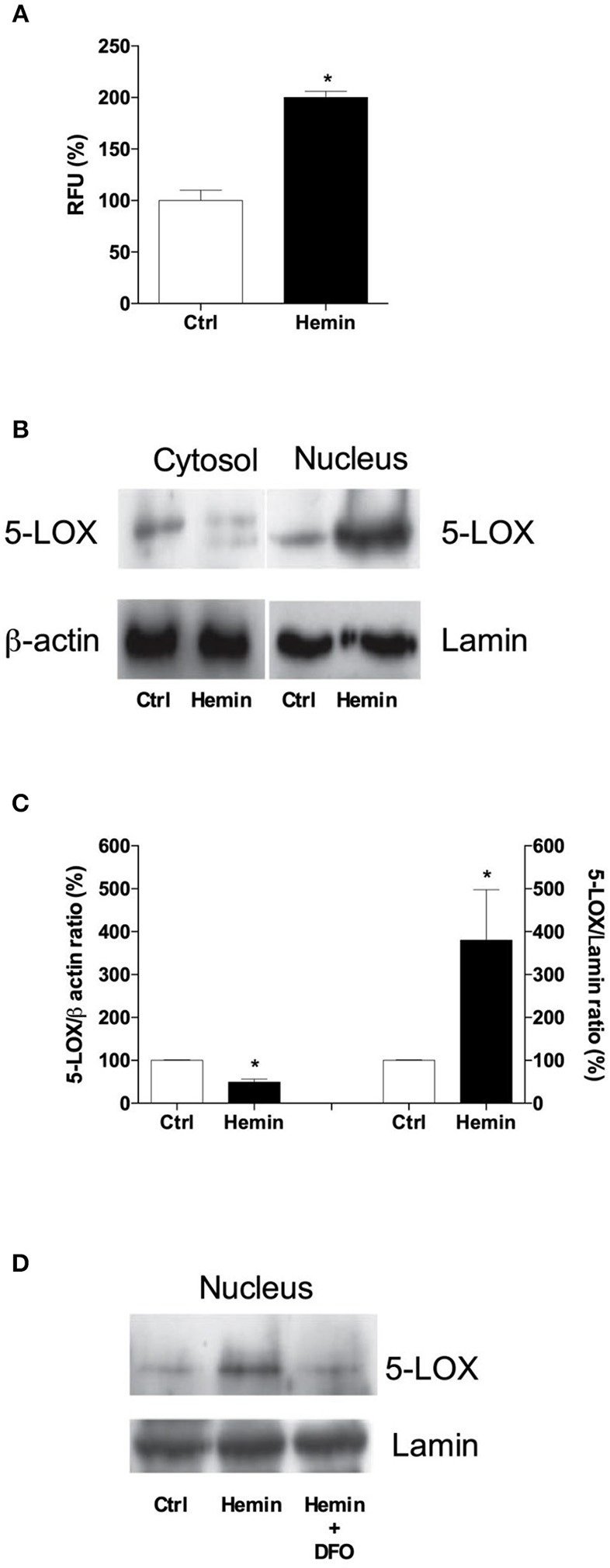
Effects of hemin administration on activity and subcellular localization of 5-LOX in THP-1 macrophages. Treatment with hemin (10 μM) for 5 min caused an overt increase in 5-LOX activity **(A)**, paralleled by a nuclear translocation of the enzyme **(B,C)**. Pre-incubation with deferoxamine (DFO, 20 μM) completely abolished the localization of 5-LOX in the nuclear fraction **(D)**. Results are given as mean ± SE of 3 independent experiments (^*^p < 0.05 vs. control). Lamin was used as a housekeeping protein for data normalization.

To investigate whether Fe^3+^ arising from heme degradation can modulate 5-LOX intracellular localization, we exposed THP-1 macrophages to free hemin. As shown in Figure 5A hemin treatment induce an increase of LOX-activity (~100%). Western blot analysis of subcellular fractions revealed that hemin, much alike Fe^3+^, produced a nuclear translocation of 5-LOX (Figures 5B,C). To test whether this effect was mediated by Fe^3+^ itself, we treated THP-1 macrophages with the Fe^3+^-chelating agent DFO, at 20 μM, for 2 h before hemin exposure. As shown in Figure 5D, DFO completely abolished the hemin-dependent translocation of 5-LOX to the nuclear fraction, underlying the pivotal role of Fe^3+^ in mediating 5-LOX activity and intracellular redistribution.

### Acute Exposure With Fe^3+^ or Hemin Regulates 5-LOX Translocation on Nuclear Envelope to Co-localize With FLAP in THP-1 Macrophages and Primary Human Macrophages

**Figure 6:**
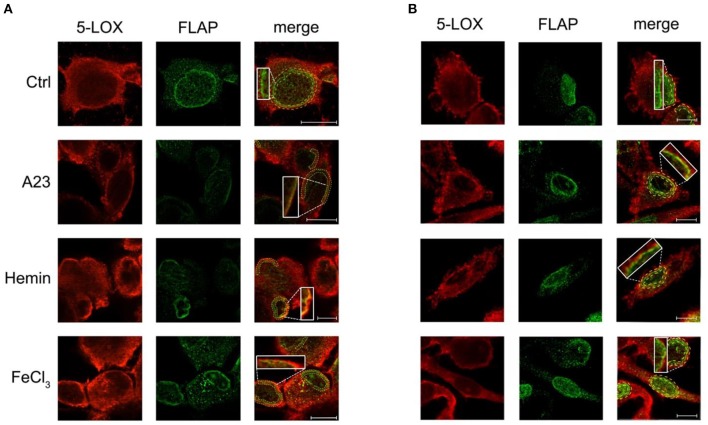
Effect of hemin or FeCl_3_ on 5-LOX subcellular distribution and its co-localization with FLAP in THP-1 cells **(A)** and human macrophages **(B)**. Cells were left untreated (Ctrl) or treated with A23187 (A23), hemin or FeCl_3_, then double stained with anti-5-LOX (red, panels on the left) and anti-FLAP (green, panels in the center). Details are given under Materials and Methods, and parameter values are summarized in Table 1. Merged images, along with specific 2X magnified regions, are shown in the panels on the right. For quantification of the overlap coefficient, the co-localization analysis of the two stainings was restricted to the region of interest which delimitates the nuclear envelop (yellow dotted lines). Images are representative of two independent experiments, for a total of 20 cells. Scale bars, 10 μm.

The effect of Fe^3+^ and hemin on the subcellular localization of 5-LOX and FLAP in THP-1 macrophages and primary human macrophages was also investigated by confocal microscopy. Fluorescence micrographs of untreated cells confirmed the presence of 5-LOX in both cytosol and nucleus, with a prevalence of the cytosolic localization, while FLAP displayed a prominent distribution in the nuclear envelope (Figures 6A,B). The Ca^2+^ ionophore A23187 was used as positive control to stimulate the translocation of 5-LOX to the nuclear envelop. Furthermore, in both cell lines exposure to Fe^3+^ or hemin induced a translocation of 5-LOX to co-localize with FLAP on nuclear envelope, the protein necessary for the enzyme activation (Figure 6 and Table 1).

**Table 1:** Effect of treatment with hemin and FeCl_3_ on co-localization of FLAP/5-LOX.

**Cells**	**Overlap coefficient**			
	**Treatment**			
	**Ctrl**	**A23**	**Hemin**	**FeCl_**3**_**
THP-1	0.07 ± 0.05	0.41 ± 0.11^***^	0.49 ± 0.19^***^	0.34 ± 0.09^***^
Primary macrophages	0.12 ± 0.08	0.75 ± 0.21^***^	0.41 ± 0.16^***^	0.42 ± 0.15^***^

*A23, A23187. Data are means S.E. values (n = 20). Significance was calculated using an one-way ANOVA with Dunnett's multiple comparisons test*.

*** *p <0.001 vs. relative Ctrl*.

### Acute Exposure With Fe^3+^ and Hemin Induces Functional Activation of THP-1 Macrophages

**Figure 7:**
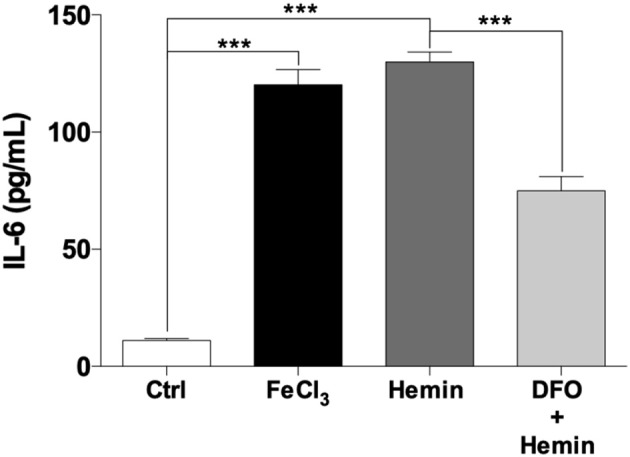
Iron, hemin, and DFO effects on IL-6 concentrations in supernatants of THP-1-derived macrophages. Effect of FeCl_3_ (10 μM for 5 min) and hemin (10 μM for 5 min) on IL-6 levels in THP-1 macrophages evaluated by ELISA. To determine the effect of exogenous iron and hemin on functional activation of macrophages, the IL-6 levels in supernatants were analyzed after 24 h by ELISA (*n* = 6). Pre-incubation with DFO (20 μM for 2 h) reduced IL-6 levels induced by hemin treatment. The results represent the means of RFU ± SE of three independent experiments (^***^p < 0.0001).

To test whether Fe^3+^ and hemin can affect the functional activation of THP-1-derived macrophages, an ELISA assay was performed to determine the expression of IL-6, a soluble cytokine that is synthesized by activated macrophages (27). The control medium from THP-1-derived macrophages presented an IL-6 concentration of 11.1 ± 0.9 pg/mL. The acute exposure of cells to Fe^3+^ and hemin significantly increased the levels of IL-6 in the supernatants showing values of 120.2 ± 6.6 and 129.9 ± 4.3 pg/mL, respectively (Figure 7). Furthermore, pre-treatment THP-1-derived macrophages with DFO leads to a significant reduction of hemin-induced IL-6 levels to 74.8 ± 6.0 pg/mL (Figure 7).

## Discussion

Increasing evidence demonstrates that iron homeostasis must be tightly regulated to maintain erythropoietic functions, redox reactions and cellular immune responses and that excessive iron levels could act as primary pro-oxidant leading to cellular damage and death (28–30). In addition, the effect of inflammation on the regulation of iron metabolism is widely recognized (31), and a dysregulated iron homeostasis or iron overload are a cornerstone of acute and chronic inflammatory processes involving cell-mediated immunity (32). According to this, macrophages play key roles in iron metabolism, in particular by recycling and storing heme iron from phagocytosed red blood cells (33, 34). Heme iron has several proinflammatory activities, including production of cytokines and acute-phase proteins, and is endowed with the ability to induce neutrophil migration and activation (35, 36). Noteworthy, it was previously reported a heme-induced biosynthesis of LTB_4_ in the nuclear membrane by the combined action of 5-LOX and LTA_4_ hydrolase (37). Moreover, it is to underline that iron-associated pro-inflammatory conditions with 5-LOX macrophage activation is intimately related to different diseases, from atherosclerosis (38), to Alzheimer's disease (39, 40), multiple sclerosis (41), and cancer (42, 43). However, the precise mechanism(s) underlying the relationship between iron and 5-LOX are as yet unclear.

In this work, combining molecular approaches with cellular and biochemical analyses, we provide clear evidence that: (*i*) the apo-form of 5-LOX obtained removing Fe^3+^ with chelators is completely inactive and shows a lower membrane binding affinity with respect to the holo-5-LOX; (*ii*) the presence of iron in the active site stabilizes an active conformation of 5-LOX more suitable for the association with membranes; (*iii*) acute treatment of macrophages with both Fe^3+^ and hemin induces a rapid translocation of 5-LOX from cytosol to nucleus leading to a specific interaction with FLAP; (*iv*) chelation of Fe^3+^ is able to revert the subcellular localization of 5-LOX; *(v)* Fe^3+^ and hemin induces a functional activation of THP-1 derived macrophages increasing levels of IL-6 and chelation of Fe^3+^ is able to significantly revert this effect.

Here we focused on the evaluation of the spontaneous membrane binding properties of 5-LOX in the presence and in the absence of iron within the active site (without other known effectors, such as Ca^2+^) and on the 5-LOX nuclear translocation only due to an acute iron (or hemin) treatment in macrophage cells. The membrane binding data reported in this study are in line with previous results obtained with LOX-1 from soybean seeds (20) and confirm a crucial general role for Fe^3+^ in preserving the structural stability and membrane binding ability of LOXs. In this context, the already reported presence of an apo-form of the enzyme in several mammalian cells (44, 45), strongly suggest a general mechanism of LOX cellular activation where excess of iron induces an enzyme translocation to the nuclear membrane and a functional interaction with FLAP, that in the case of 5-LOX is a necessary prerequisite for the pro-inflammatory LTs biosynthesis (15, 16). Indeed, we demonstrated that Fe^3+^ and hemin promote the co-localization of 5-LOX and FLAP in the nuclear envelope of both THP-1 and human macrophages that is accompanied with an overexpression of IL-6 in THP-1 derived macrophages (Figure 7).

In general, the regulatory mechanisms that facilitate the transient activation of enzymes like 5-LOX may include modulation of their transcription and/or translation, targeted degradation of the protein, phosphorylation, and/or allosteric control of their catalytic activity (15, 16, 46). On the basis of the present data, we can speculate the presence of a fraction of 5-LOX in the apo-form in human macrophages. Thus, being the biosynthesis and maintenance of a catalytically inactive apo-5-LOX an event energetically unfavorable for a cell, our results suggest that this apo-forms may function as “stand by” inactive forms able to readily incorporate Fe^3+^ in the active site, and thus to rapidly respond to specific physiological or pathological cellular stimuli. In this way, 5-LOX activity could be readily increased post-translationally without waiting for (slower) transcriptional and/or translational processes. To our knowledge, as yet only one apo-enzyme has been reported to be activated by an immediate post-translational mechanism, namely human Cu, Zn-superoxide dismutase (47).

As above discussed, our study clearly evidenced an iron-induced mechanism of 5-LOX activation that could have a physiological relevance but it could be also related to an iron overload condition occurring with iron supplementation or excess of bioavailable iron in the diet. Indeed, from a clinical point of view, our results suggest a more careful evaluation of the already evidenced risks related to iron overloading and supplementation reported by different iron intake recommendations (e.g., US Food and Nutrition Board, FAO/WHO, and the EU Scientific Committee). In line with this, our study underlines the importance of using different clinical biomarkers (e.g., the ferritin plasma levels, the apo- and holo-transferrin ratio, the apo-heme concentration in erythrocytes, the mean corpuscular volume, etc.) for a proper assessment of iron deficiency—and thus anemia—before administering a therapy of iron supplementation that here we are speculating that could be associated to a chronic activation of macrophages, possibly explaining the already described risks of iron overload (48) and linked to inflammatory-related diseases (38–43, 49).

As a final note, Fe^3+^ chelators, such as the FDA-approved drugs deferiprone or DFO, have been shown to inhibit the progression and the proliferation of cancer cells through a variety of mechanisms such as the inhibition of iron-dependent activation of translational and enzymatic processes (50–53). Moreover, it has been shown that the iron content of macrophages affects the associated infiltration capacity; the high-Fe^3+^ macrophages are the most able to infiltrate the tumor compared to the general macrophage populations found in the tumor (54). In fact, recruitment of tumor-associated macrophages (TAMs) -usually associated with advanced tumor progression and metastasis- is one of the key events in tumor and a correlation between 5-LOX and FLAP levels and the density of TAMs has been found in ovarian cancer (55, 56). The functional effects that we here reported using DFO suggest that the therapeutic potential of iron chelators could be due, at least in part, by modulating the cellular distribution and activity of 5-LOX.

## Conclusions

Taken together, these results indicate that iron modulates 5-LOX intracellular localization by increasing the ability of the enzyme to bind to nuclear membranes thus activating the 5-LOX-mediated inflammatory processes. Our data also identify a potentially important mechanism regarding the role of 5-LOX in the functional activation of macrophages, and may advance our understanding of the risks associated to iron overloading.

## Data Availability

The raw data supporting the conclusions of this manuscript will be made available by the authors, without undue reservation, to any qualified researcher.

## Author Contributions

BD and AD performed cell cultures, molecular biology studies, subcellular fractionation, localization analyses and BD and A-KH did functional studies. CA performed membrane binding experiments and enzyme measurements. LS and SO performed cell isolation and cultures and confocal analyses. MM and ED conceived the project. AD, BD, SO, MS, MM, and ED analyzed the data and wrote the paper with relevant inputs from all co-authors.

### Conflict of Interest Statement

The authors declare that the research was conducted in the absence of any commercial or financial relationships that could be construed as a potential conflict of interest.

## Supplementary Material

Supplementary Figure 1 SDS-PAGE analysis of human 5-LOX purification. Lane M: Protein molecular weight standards; lane 1: Cell extracts of *E. coli* transformed with plasmid pT3-5-LOX after ATP-agarose purification.

## Abbreviations

LOXs: lipoxygenases
FLAP: 5-LOX activating protein
LT: leukotrienes
PMA: phorbol-12-myristate-13-acetate
AA: arachidonic acid
NDGA: nordihydroguaiaretic acid
DFO: deferoxamine
FBS: fetal calf serum
FRET: fluorescence resonance energy transfer
cPLA2: cytosolic phospholipase A2
ROS: reactive oxygen species
Hb: hemoglobin.
