# Supplementary figures and images for: Iron-Dependent Trafficking of 5-Lipoxygenase and Impact on Human Macrophage Activation
Source: Front Immunol. 2019 Jun 28;10:1347. doi: 10.3389/fimmu.2019.01347 (PMC6610208; doi:10.3389/fimmu.2019.01347)

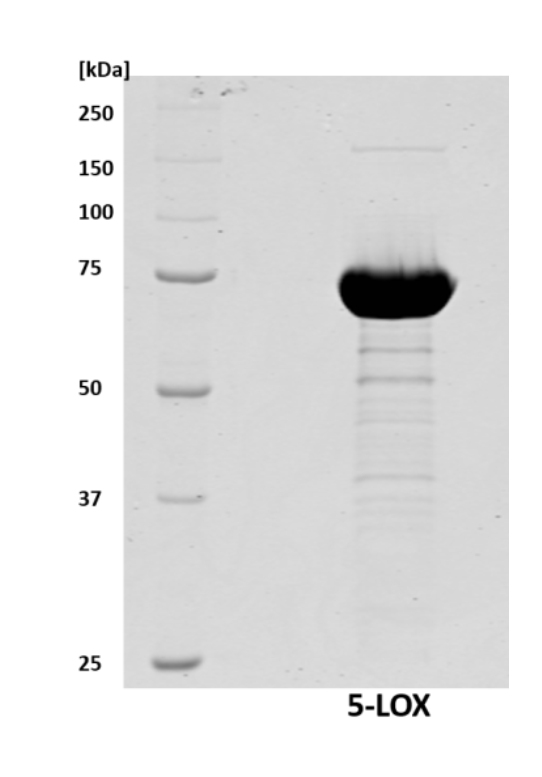

Supplement: Supplementary Figure 1 — SDS-PAGE analysis of human 5-LOX purification. Lane M: Protein molecular weight standards; lane 1: Cell extracts of E. coli transformed with plasmid pT3-5-LOX after ATP-agarose purification. [file Image_1.TIFF]
